# Optimal programmed intermittent epidural bolus volume for dural puncture epidural labor analgesia in patients with gestational hypertension: a biased-coin up-and-down sequential allocation trial

**DOI:** 10.3389/fmed.2025.1719334

**Published:** 2025-12-09

**Authors:** Binghui Zhang, Hongyang Zhang, Yuan Wu, Kai Zhao, Yancen Meng

**Affiliations:** 1 Department of Anesthesiology, The Fourth Hospital of Shijiazhuang, Shijiazhuang, China; 2 Department of Obstetrics, The Fourth Hospital of Shijiazhuang, Shijiazhuang, China

**Keywords:** analgesia, epidural, anesthesia, spinal, obstetrical, infusions, hypertension, pregnancy-induced

## Abstract

**Background:**

This study aimed to evaluate the effective programmed intermittent epidural bolus (PIEB) volume and the efficacy of dural puncture epidural (DPE) for labor analgesia in patients with gestational hypertension (GH).

**Methods:**

Fifty primiparous women with GH, aged 22–40 years and classified as American Society of Anesthesiologists physical status II, scheduled for DPE labor analgesia were included. A biased-coin up-and-down sequential method was used to determine bolus volumes (7–12 mL). The first patient received 7 mL, with subsequent adjustments based on the need for rescue analgesia within 6 h of initiation or full cervical dilation. The truncated Dixon and Mood method and isotonic regression analysis were employed to calculate the 90% effective PIEB volume (EV90) and 95% confidence interval (CI). Recorded parameters included maximum sensory and motor blockade scores, mean arterial pressure, adverse events, and neonatal outcomes (umbilical artery pH, Apgar scores at 1 and 5 min).

**Results:**

The EV90 was 9.82 (95% CI: 9.41–10.23) mL using the truncated Dixon and Mood method and 9.95 (95% CI: 9.52–10.38) mL using isotonic regression. The incidence of sensory blockade ≥T6 increased with higher volumes: 0% (7–9 mL), 13.33% (10 mL), 25.00% (11 mL), and 60.00% (12 mL). In the 12 mL group, one case of Bromage grade 1 motor blockade (recovered within 6 h), one of fetal bradycardia (resolved after maternal repositioning), and two cases of hypotension not requiring treatment were observed. No significant intergroup differences were detected in the duration of the first or second stage of labor or in the use of antihypertensive medications (*p* > 0.05).

**Conclusion:**

For patients with GH receiving DPE with PIEB (40-min interval; 0.08% ropivacaine + 0.3 μg/mL sufentanil), the EV90 was 9.89 mL. This regimen significantly reduced the risk of excessive sensory block and hypotension while ensuring effective analgesia.

**Clinical trial registration:**

www.chictr.org.cn, identifier ChiCTR2500099107.

## Introduction

Hypertensive disorders of pregnancy, which affect approximately 5%–10% of all pregnancies, are among the most common obstetric complications and represent one of the leading causes of maternal and perinatal mortality (1). Patients with gestational hypertension (GH) exhibit pathophysiological changes such as systemic arteriolar spasm and vascular endothelial injury. In these patients, the physiological stress induced by labor pain stimulates increased secretion of hormones, such as catecholamines, which may exacerbate hemodynamic instability and thereby increase perinatal risks (2). Therefore, achieving an optimal balance between effective analgesia and hemodynamic stability in this high-risk population remains a critical challenge in obstetric anesthesia.

Neuraxial analgesia is widely recommended for labor analgesia (3, 4). However, traditional epidural anesthesia has limitations, including slow onset and a high incidence of inadequate analgesia (5, 6). These shortcomings may induce or exacerbate the maternal stress response in patients with GH, thereby increasing perinatal risks. Recent studies indicate that the dural puncture epidural (DPE) technique combined with programmed intermittent epidural bolus (PIEB) can significantly improve the quality of labor analgesia (7, 8). DPE involves puncturing the dural mater to allow local anesthetics to act directly on the subarachnoid space, leading to a rapid onset of analgesia (9, 10). PIEB delivers intermittent boluses to maintain a stable analgesic level, reducing breakthrough pain while minimizing motor blockade (11, 12). However, the efficacy of PIEB is highly dependent on specific parameter settings, including the type and concentration of drugs, bolus volume, dosing interval, and infusion rate. While previous studies have investigated optimal bolus volumes for conventional epidural labor analgesia and provided relevant clinical recommendations, these guidelines are largely based on findings in healthy parturients. Evidence regarding the impact of PIEB parameters on both analgesic efficacy and hemodynamic stability in patients with GH remains limited (13, 14).

Therefore, we hypothesized that for patients with GH receiving DPE combined with PIEB, an optimal PIEB bolus volume exists that maximizes analgesic efficacy while minimizing hemodynamic perturbations. The primary objective of this biased-coin up-and-down sequential allocation trial was to identify the 90% effective volume (EV90) for PIEB in this specific high-risk population, aiming to provide an evidence-based foundation for safer and more effective labor analgesia.

## Methods

This study was approved by the Medical Ethics Committee of the Fourth Hospital of Shijiazhuang (Approval No. 20250066) and registered with the Chinese Clinical Trial Registry on March 18, 2025 (Registration No. ChiCTR2500099107). The trial was conducted in accordance with the Declaration of Helsinki. All participants provided written informed consent before enrollment. A total of 50 nulliparous women with GH scheduled to receive labor analgesia were recruited. Eligible participants were aged 22–40 years. This age range was chosen to reflect the typical childbearing population in our center and to minimize potential physiological and psychological confounders associated with younger adulthood, which may influence pain perception and labor progression. Additional inclusion criteria were a singleton term pregnancy (gestational age ≥37 weeks), a BMI ≤ 35 kg/m^2^, and were classified as American Society of Anesthesiologists (ASA) physical status II. The diagnosis of GH followed the 2019 American College of Obstetricians and Gynecologists (ACOG) guidelines (15). Exclusion criteria included ① history of allergy to local anesthetics or opioids, ② use of analgesic medications within 4 h before delivery, ③ concurrent acute or chronic infectious diseases, ④ comorbid autoimmune, hematologic, or psychiatric disorders, and ⑤ platelet count <100 × 10^9^/L or any contraindication to neuraxial anesthesia. Withdrawal criteria included ① occurrence of severe adverse reactions, ② unintended dural puncture with the epidural needle, ③ absence of cerebrospinal fluid (CSF) outflow upon dural puncture with the spinal needle, or ④ voluntary withdrawal by the patient from the clinical trial.

When cervical dilation reached 2–3 cm, patients were transferred to the labor analgesia room. A peripheral venous line was established, and a compound sodium chloride solution was infused at 10 mL·kg^−1^·h^−1^. Maternal vital signs and fetal heart rate (FHR) were monitored continuously. Patients were positioned in the left lateral decubitus position. Under ultrasound guidance, the epidural space was identified at the L2–3 interspace with a 17G Tuohy needle using the loss-of-resistance-to-air technique. Following successful puncture, the epidural catheter was not immediately inserted. Instead, a 25G Whitacre spinal needle was advanced through the Tuohy needle to penetrate the dura mater. After confirmation of CSF reflux, the Whitacre needle was withdrawn, and an epidural catheter was inserted cephalad 3–4 cm into the epidural space. After negative aspiration for CSF or blood, the catheter was secured. The patient was then placed in the supine position with left uterine displacement, and the head of the bed was elevated to 30°. A test dose of 3 mL of 1.5% lidocaine was administered. Following a 5-min observation period without any adverse reactions, a loading dose of 12 mL of 0.08% ropivacaine combined with 0.3 μg/mL sufentanil was administered over 2 min. Patients who achieved a Visual Analogue Scale (VAS; 0 mm = no pain, 100 mm = unbearable pain) score ≤30 mm within 20 min after the completion of the loading dose were considered eligible to continue in the study.

The epidural pump was connected 40 min after the initial bolus. The analgesic solution consisted of a 100-mL mixture of 0.08% ropivacaine and 0.3 μg/mL sufentanil. The pump was programmed with a bolus interval of 40 min, a PCEA bolus of 5 mL, a lockout time of 20 min, and a maximum hourly dose of 30 mL. Tested PIEB bolus volumes ranged from 7 to 12 mL. This range was selected based on previous dose-finding studies and randomized controlled trials investigating PIEB for labor analgesia, which commonly employed effective volumes within this spectrum (16, 17). Successful analgesia was defined as not needing PCEA use or manual rescue bolus within 6 h of initiation or until full cervical dilation, whichever occurred first. The first patient received a bolus volume of 7 mL. For subsequent patients, bolus volume was determined by the response of the previous participant. A successful response (no PCEA or rescue intervention) was followed by the next patient being assigned, according to a random response list generated by a statistician using Excel, to either a 1/9 probability of a 1-mL decrease or an 8/9 probability of the same volume. An unsuccessful response resulted in the next patient’s bolus volume being increased by 1 mL. If analgesia was adequate at 7 mL or inadequate at 12 mL, the subsequent bolus volume remained unchanged. Using this biased-coin up-and-down sequential design, patients were sequentially assigned to bolus volumes of 7, 8, 9, 10, 11, or 12 mL. To maintain blinding, the infusion pump screen was covered with opaque tape throughout the study. Both investigators and participants remained blinded to the programmed bolus volume until completion of data analysis.

Management of adverse events:

The management of adverse events was strictly governed by a predefined protocol to ensure patient safety throughout the study.

(1) Immediate test dose reaction: After the administration of the 3 mL test dose of 1.5% lidocaine, patients were observed for 5 min. Signs of local anesthetic systemic toxicity (e.g., tinnitus, perioral numbness) or total spinal anesthesia (e.g., rapidly occurring hypotension, loss of consciousness) would lead to immediate cessation of the procedure and management according to established resuscitation protocols. The affected participant would then be excluded from the study.

(2) Adverse events during labor analgesia:

Hypotension: If systolic blood pressure decreased by more than 20% from baseline and did not respond to positional change or fluid administration, 50 μg of intravenous phenylephrine was administered as the first-line vasopressor. If bradycardia coexisted with hypotension, 5 mg of intravenous ephedrine was available as an alternative.

Hypertension: If systolic blood pressure increased by more than 20% above baseline, 12.5 mg of intravenous urapidil was administered.

Bradycardia: For maternal bradycardia (heart rate <50 beats/min), 0.5 mg of intravenous atropine was administered, with repeat dosing as needed.

Fetal heart rate decelerations: Upon detection of fetal heart rate decelerations, initial management included maternal repositioning and supplemental oxygen. If the abnormality persisted, further interventions were guided by standard obstetric protocols.

Other opioid-related events: For other adverse events such as pruritus, nausea, or vomiting, mild cases were recorded and monitored. If the symptoms were severe or intolerable to the patient, symptomatic pharmacological treatment was provided.

The primary outcome was the incidence of effective analgesia, defined as no requirement for a PCEA or manual medication administration within 6 h after initiation of labor analgesia, or until full cervical dilation, whichever occurred first. The selection of a 6-h observation window was based on practicality and standardization considerations, aiming to evaluate the sustained efficacy of this PIEB regimen during the first stage of labor. This timeframe aligns with previous studies employing a tilted coin design to investigate PIEB parameters (18, 19).

Secondary outcomes included:

(1) Sensory and motor block: Assessed by an investigator not involved in the study protocol at 20 and 60 min after the loading dose, and subsequently every hour. The upper level of sensory blockade was determined using the cold test, and the degree of motor blockade was evaluated using the modified Bromage scale (Grade 0: no motor block; Grade 1: inability to raise the extended leg; Grade 2: inability to flex the knee; Grade 3: inability to flex the ankle joint).
(2) Hemodynamic parameters: Mean arterial pressure (MAP) was recorded every 20 min until the end of the study period.
(3) Labor duration and neonatal outcomes: The durations of the first and second stages of labor were recorded. Neonatal outcomes were assessed via Apgar scores at 1 and 5 min after birth and umbilical artery blood gas analysis.
(4) FHR monitoring: FHR was continuously monitored via cardiotocography from 30 min before analgesia initiation until delivery. Abnormalities were defined as: (a) late decelerations: gradual, symmetrical decreases in FHR, with nadir occurring after the peak of uterine contraction, and requiring ≥30 s from onset to nadir; (b) variable decelerations: abrupt decreases in FHR ≥ 15 bpm below baseline, lasting 15 s to 2 min, with onset to nadir ≥30 s; (c) prolonged decelerations: decreases in FHR ≥ 15 bpm below baseline, lasting 2–10 min; and (d) tachycardia (baseline FHR > 160 bpm) or bradycardia (baseline FHR < 110 bpm) persisting for >10 min.
(5) Other adverse events: Documented events included nausea and vomiting, dramatic fluctuations in blood pressure (systolic variation >20% from baseline), bradycardia (HR < 50 bpm), tachycardia (HR > 120 bpm), pruritus, post-dural puncture headache, eclampsia, and the use of antihypertensive drugs.

### Statistical analysis and sample size

The biased-coin up-and-down sequential method was employed with a pre-set probability bias ratio (*b*). This design is particularly suitable for dose-finding studies with limited sample sizes. It preferentially allocates participants to doses near the anticipated effective level, thereby maximizing the information obtained from each sequential patient. To analyze data from this design, we used a two-step analytical approach. First, we applied the truncated Dixon and Mood method to provide a preliminary EV90 estimate. Next, we performed isotonic regression analysis to refine this estimate by imposing a monotonic dose–response relationship, thereby enhancing the validity and precision of the final EV90 value. This combined approach is widely used in anesthetic dose-finding studies and is well-suited for the biased-coin up-and-down design. As this was an exploratory study with non-independent data and an unknown prior distribution, we referred to simulation studies by Stylianou et al., which suggest that 20–40 participants are sufficient to obtain a stable estimate of the effective interval (20, 21). Accounting for potential dropouts, we initially enrolled 50 patients; ultimately, 44 patients completed the trial and were included in the final analysis.

Statistical analyses were performed using R software (version 4.1.0) and SPSS (version 26.0; IBM, Armonk, NY, USA). The 90% effective bolus volume (EV90) and its 95% confidence interval (CI) were calculated using the truncated Dixon and Mood method combined with isotonic regression analysis. Continuous variables with a normal distribution are presented as mean ± standard deviation (SD) and were compared using one-way ANOVA, with post-hoc pairwise comparisons conducted using the Student–Newman–Keuls test. Non-normally distributed continuous variables are presented as median (interquartile range, IQR) and were compared using the rank-sum test. Categorical variables are presented as percentages (%). A *p*-value < 0.05 was considered statistically significant.

## Results

A total of 50 patients with GH were initially enrolled. Six patients were excluded: two with VAS scores >30 mm at 20 min after the initial loading dose, one owing to epidural catheter dislodgement during the procedure, two because of inadvertent intravascular epidural catheter placement, and one in whom CSF leakage was not observed after removal of the Whitacre needle. Ultimately, 44 patients completed the study and were included in the analysis. Demographic characteristics (age, gestational age and BMI) and obstetric baseline indicators are summarized in Table 1.

**Table 1:** Demographic characteristics and baseline parameters of patients with GH.

Characteristic	Value (Mean ± SD)
Gestational Age (weeks)	38.83 ± 0.99
Age (years)	27.41 ± 3.30
Height (cm)	163.77 ± 4.14
Weight (kg)	82.19 ± 11.17
BMI (kg/m^2^)	30.62 ± 3.81
Baseline fetal heart rate (bpm)	140.07 ± 2.62
VAS pain score at analgesia initiation (mm)	81.75 ± 4.62
Cervical dilation at analgesia initiation (cm)	2.43 ± 0.50
Mean arterial pressure (MAP) at analgesia initiation (mmHg)	108.45 ± 2.10

Statistical tests: data are presented as mean (SD).

BMI, body mass index; bpm, beats per minute; VAS, Visual Analogue Scale. VAS 0–100 mm, where zero = no pain and 100 mm = worst pain imaginable.

**Figure 1:**
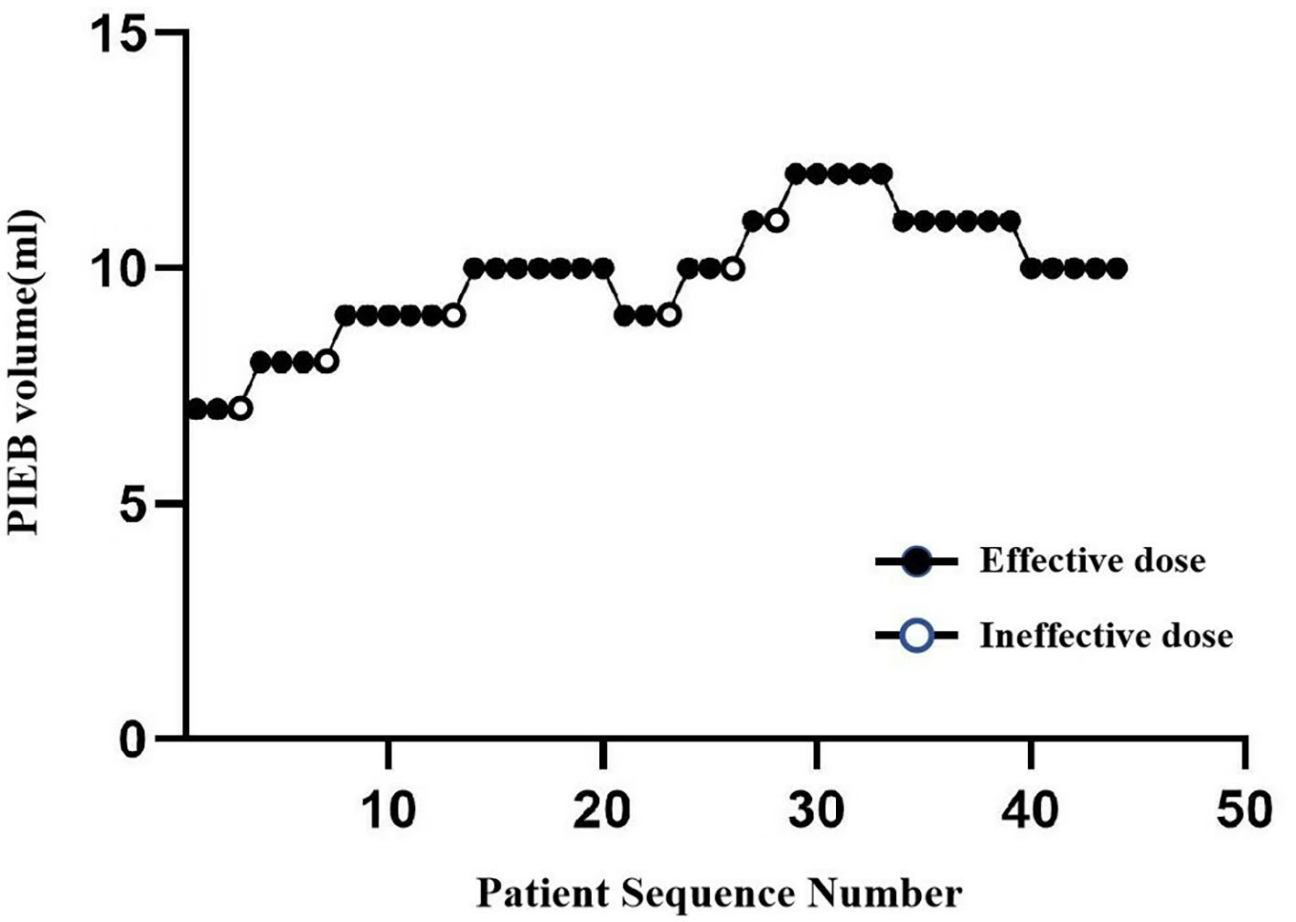
Allocation sequence and response patterns to the assigned PIEB pulse volumes in patients with GH. The patient with GH sequence number (*x*-axis) is ordering of patient exposures using the bias coin up-and-down design. The assigned PIEB volume (*y*-axis) are 7, 8, 9, 10, 11, and 12 mL. An effective PIEB volume is denoted by a filled circle, while an ineffective one is denoted by an open circles.

The allocation sequence and response outcomes for each PIEB bolus volume group are shown in Figure 1. The estimated EV90 was 9.82 (95% CI: 9.41–10.23) mL using the truncated Dixon–Mood method and 9.95 (95% CI: 9.52–10.38) mL using isotonic regression analysis. Using a weighted average algorithm, the overall EV90 of DPE for labor analgesia in this population was 9.89 mL. The observed response rates for each PIEB bolus volume, along with the response rates adjusted using the pooled adjacent violators algorithm (PAVA), are summarized in Table 2.

**Table 2:** Response rates and PAVA-adjusted response rates to various PIEB pulse volumes in patients with GH.

PIEB pulse volume (mL)	Number of patients (*n*)	Number of successes/total patients	Observed response rate (%)	PAVA-adjusted response rate (%)
7	3	2/3	66.7	66.7
8	4	3/4	75	75
9	9	7/9	77.8	77.8
10	15	14/15	93.3	93.3
11	8	7/8	87.5	87.5
12	5	5/5	100	100

Statistical tests: PAVA-adjusted response rates were estimated by the use of a weighted isotonic regression method.

PAVA, pool-adjacent-violators algorithm.

A total of six patients required rescue analgesia owing to inadequate pain relief; their details are provided in Table 3.

**Table 3:** Failure time of PIEB and time from analgesia initiation to PCEA bolus in patients with GH.

Patient number	PIEB pulse volume (mL)	Time from analgesia to PCEA bolus (min)
3	7	145
7	8	180
13	9	135
23	9	155
26	10	150
28	11	90

PIEB, programmed intermittent epidural bolus; PCEA, patient-controlled epidural analgesia.

The incidence of a sensory blockade level ≥T6 in each group was as follows: 7 mL group: 0% (0/3); 8 mL group: 0% (0/4); 9 mL group: 0% (0/9); 10 mL group: 13.33% (2/15); 11 mL group: 25.00% (2/8); and 12 mL group: 60.00% (3/5). Motor blockade (Bromage grade 1) was observed in only one patient in the 12 mL group. The overall incidence of adverse events was 11.36% (5/44). Reported events included vomiting (one patient in the 11 mL group), pruritus (one patient in the 12 mL group), fetal bradycardia (one patient in the 12 mL group), and hypotension (two patients in the 12 mL group). No cases of eclampsia were noted, and no patient required antihypertensive medication. The results are summarized in Table 4.

**Table 4:** Sensory block level, degree of motor block, and adverse events in patients with GH.

Group	Number of patients (*n*)	Sensory block level (number of patients)							Sensory block level (number of patients)		Adverse events (number of patients)			
		T4	T5	T6	T7	T8	T9	T10	0	1	Vomiting	Pruritus	Fetal Bradycardia	Hypotension
7 ml	3	0	0	0	0	0	1	2	3	0	0	0	0	0
8 ml	4	0	0	0	1	1	1	1	4	0	0	0	0	0
9 ml	9	0	0	0	3	4	2	0	9	0	0	0	0	0
10 ml	15	0	0	2	5	7	0	1	15	0	0	0	0	0
11 ml	8	0	0	2	3	3	0	0	8	0	1	0	0	0
12 ml	5	0	1	2	2	0	0	0	4	1	0	1	1	2

A significant decrease in MAP was observed in all patients after the initiation of labor analgesia (*p* < 0.05). However, no statistically significant differences in MAP were found among the groups at any time point (*p* > 0.05). The results are summarized in Table 5. Similarly, there were no significant intergroup differences in the duration of the first or second stage of labor or in umbilical artery pH values (*p* > 0.05). The results are summarized in Table 6. All neonates received Apgar scores of 10 at both 1 and 5 min after birth.

**Table 5:** Comparison of MAP at different time points among groups of patients with GH.

Group	MAP before analgesia	20 min MAP	60 min MAP	2 h MAP	3 h MAP	4 h MAP	5 h MAP	6 h MAP	*F*_1_value	*P*_1_value
7 ml	107.00 ± 2.00	98.67 ± 2.08	102.00 ± 2.00	99.00 ± 1.73	100.00 ± 2.65	103.00 ± 0.00	98.00 ± 0.00	99.00 ± 0.00	2.366	0.026
8 ml	110.75 ± 0.50	101.00 ± 1.83	102.75 ± 4.50	101.50 ± 2.38	101.50 ± 5.07	100.25 ± 4.19	100.75 ± 2.87	100.25 ± 2.06	2.566	0.012
9 ml	107.67 ± 2.06	100.56 ± 2.88	101.00 ± 2.74	102.38 ± 2.72	101.88 ± 2.53	100.43 ± 1.72	100.33 ± 3.51	99.50 ± 2.12	2.166	0.033
10 ml	109.13 ± 1.81	101.27 ± 2.19	101.27 ± 2.49	100.83 ± 2.04	101.30 ± 2.67	101.11 ± 1.45	101.38 ± 1.92	100.60 ± 2.41	3.366	0.006
11 ml	107.63 ± 2.50	100.75 ± 2.71	100.38 ± 1.51	100.13 ± 1.36	103.13 ± 2.23	101.00 ± 2.00	100.83 ± 2.48	101.17 ± 3.60	3.121	0.011
12 ml	108.20 ± 1.64	100.80 ± 2.28	100.20 ± 0.84	101.00 ± 1.41	101.75 ± 1.26	102.00 ± 3.16	102.00 ± 1.41	101.50 ± 2.12	2.968	0.047
*F*_2_value	2.429	0.597	0.716	1.658	0.690	0.465	0.456	0.230		
*P*_2_value	0.053	0.702	0.615	0.171	0.635	0.798	0.803	0.943		

Statistical tests: data are presented as ANOVA.

**Table 6:** Comparison of the first stage duration, second stage duration, and umbilical artery blood gas pH value among groups of patients with GH.

Group	First stage duration (min)	Second stage duration (min)	Umbilical artery pH
7 ml	365.00 ± 155.00	55.67 ± 8.74	7.28 ± 0.03
8 ml	746.25 ± 103.39	67.25 ± 56.99	7.27 ± 0.09
9 ml	419.44 ± 201.45	42.33 ± 12.19	7.30 ± 0.06
10 ml	430.80 ± 237.68	49.27 ± 33.91	7.27 ± 0.07
11 ml	583.75 ± 196.54	71.38 ± 38.41	7.27 ± 0.07
12 ml	471.00 ± 200.89	76.60 ± 31.00	7.30 ± 0.05
*F/H*	2.232	1.256	0.392
*p* value	0.071	0.303	0.851

Statistical tests: data are presented as ANOVA.

## Discussion

This prospective, double-blind trial using a biased-coin up-and-down sequential allocation design demonstrated that, for labor analgesia with DPE combined with PIEB (with a fixed bolus interval of 40 min and a solution of 0.08% ropivacaine plus 0.3 μg/mL sufentanil), the effective bolus volume required to achieve successful analgesia in 90% of patients with GH (EV90) was 9.89 mL. The incidence of high sensory block (≥T6) increased with higher bolus volumes, with the respective rates being 0% (0/3) in the 7 mL group, 0% (0/4) in the 8 mL group, 0% (0/9) in the 9 mL group, 13.33% (2/15) in the 10 mL group, 25.00% (2/8) in the 11 mL group, and 60.00% (3/5) in the 12 mL group. In the 12 mL group, adverse events included one case of fetal bradycardia (resolved after maternal positional change), and two cases of hypotension (not requiring pharmacological intervention). No significant differences were observed across the different PIEB bolus volume groups (7–12 mL) in the duration of the first or second stage of labor, use of antihypertensive medications, incidence of eclampsia, or neonatal Apgar scores at 1 and 5 min.

Hypertensive disorders of pregnancy are a major risk factor for adverse perinatal outcomes. The characteristic hemodynamic instability in this population poses a challenge for conventional analgesic regimens: sympathetic blockade induced by local anesthetics may exacerbate blood pressure fluctuations, while inadequate analgesia-induced stress responses can further increase vascular resistance, potentially compromising placental perfusion and increasing the risk of fetal hypoxia. Therefore, precisely titrating PIEB parameters to balance effective analgesia with circulatory stability remains a critical concern in obstetric anesthesia.

Our findings indicate that under fixed 40-min bolus intervals using an analgesic solution of 0.08% ropivacaine combined with 0.3 μg/mL sufentanil, the EV90 of DPE for labor analgesia in patients with GH was 9.89 mL. Similarly, Ran et al. (16), using epidural labor analgesia with a 40-min bolus interval and a biased-coin up-and-down sequential design, estimated an optimal bolus volume of 9 mL for 0.1% ropivacaine with 0.5 μg/mL sufentanil, reporting low incidences of motor blockade and adverse events. It is worth noting that although the DPE technique is thought to accelerate analgesic onset by facilitating local anesthetic transfer into the subarachnoid space through a dural puncture-induced pressure gradient (22, 23), our findings suggest that DPE did not significantly alter the optimal bolus volume requirement in patients with GH. Maeda et al. (24) reported that DPE required less bupivacaine than epidural analgesia for effective initial analgesia using a biased-coin design, yet this reduction was not reflected in maintenance bolus volume requirements. Similarly, Song et al. (25), using DPE combined with PIEB (0.1% ropivacaine + 0.3 μg/mL sufentanil, 40-min interval), estimated an EV90 of 9.2 (95% CI: 8.5–9.9) mL, closely aligning with our result. Xiao et al. (17) also reported comparable optimal PIEB volumes between DPE and traditional epidural techniques, and Yao et al. (26) observed no significant advantage of DPE over the standard epidural technique when analgesia was maintained with PIEB. These findings collectively suggest that the DPE technique, compared to the traditional epidural technique, does not appear to markedly influence the required maintenance PIEB bolus volume. Bernards et al. (27), using an *in vitro* primate dural model, demonstrated that the flux of local anesthetics into the subarachnoid space depends on the size of the dural perforation, drug characteristics, and the distance between the dural hole and the catheter tip. Correspondingly, Cappiello et al. (22) reported enhanced analgesic efficiency with a 25G Whitacre needle combined with high-volume, low-concentration local anesthetics. Based on this evidence, we used a 25G Whitacre needle for dural puncture in our study. The transfer of medication through the dural defect is influenced not only by needle gauge but also by the distribution and penetrative capacity of the drug within the epidural space. These factors are particularly critical for patients with GH, as these patients exhibit systemic small artery spasm and vascular endothelial injury, which may lead to hemodynamic instability. Such patients require effective analgesia while minimizing hemodynamic fluctuations to avoid severe hemodynamic changes, which could compromise uteroplacental perfusion and increase the risk of fetal hypoxia. To achieve this balance, we used a low concentration of ropivacaine combined with sufentanil. Ropivacaine is widely used in labor analgesia owing to its long duration of action and favorable cardiac safety profile (28). Sufentanil, a potent *μ*-opioid receptor agonist, exhibits synergistic effects with ropivacaine, leading to faster onset and prolonged duration of analgesia (29).

Our results demonstrated a positive correlation between the administered bolus volume and the incidence of high sensory blockade. The proportion of patients achieving a sensory block level at or above T6 was 0% (0/3) in the 7 mL group, 0% (0/4) in the 8 mL group, 0% (0/9) in the 9 mL group, 13.33% (2/15) in the 10 mL group, 25.00% (2/8) in the 11 mL group, and 60.00% (3/5) in the 12 mL group. This finding is consistent with previous reports: Zakus et al. (30) observed that higher bolus volumes (10–12 mL) were associated with more extensive sensory blockade compared to lower volumes (7–9 mL), and Xiao et al. (17) also reported a positive correlation between PIEB bolus volume and sensory block level during maintenance of labor analgesia. These findings suggest that larger bolus volumes may lead to a higher sensory blockade level. A potential explanation is that larger bolus volumes generate increased epidural pressure, which may promote the leakage of local anesthetics through the dural puncture site into the subarachnoid space, thereby enhancing cephalad spread and resulting in a higher sensory block. Notably, only one patient in the 12 mL group developed Bromage grade 1 motor blockade, which resolved within 6 h of catheter removal. Additionally, one case of pruritus occurred in the 12 mL group and one case of vomiting in the 11 mL group. A general downward trend in mean MAP was observed after analgesia initiation across all groups. Two patients experienced transient hypotension, both of which were successfully managed with positional adjustment without requiring pharmacological intervention. Patients with GH may be susceptible to hemodynamic instability owing to potential impairment of autonomic regulation, which could affect placental perfusion and increase the risk of fetal hypoxia. In this study, one case of fetal bradycardia was detected in the 12 mL group, which normalized after maternal repositioning. No significant differences were observed among the groups in the duration of the first or second stage of labor, use of antihypertensive medications, incidence of eclampsia, or neonatal Apgar scores at 1 and 5 min. Although higher bolus volumes were not associated with adverse neonatal outcomes in this cohort, they were linked to an increased risk of excessively high sensory blockade, abnormal FHR patterns, and hypotension in patients with GH. Based on the estimated EV90 and the profile of adverse events, we conclude that a bolus volume of 9.89 mL—using a fixed 40-min interval and an analgesic solution of 0.08% ropivacaine combined with 0.3 μg/mL sufentanil—can achieve an optimal balance between effective analgesia and hemodynamic stability in these patients. Adoption of this optimized regimen may help reduce the incidence of eclamptic seizures and other cardiocerebrovascular complications in mothers, while lowering the risk of fetal distress.

This study has several limitations. First, the observation period was limited to 6 h after analgesia initiation. We acknowledge that this may not cover the entire first stage for all parturients. This could introduce bias and limit the generalizability of our findings to the complete labor experience. Future studies designed to assess the performance of this optimized regimen throughout the entire course of labor are needed. Although the sample size was adequate for estimating the primary outcome, it may have been underpowered to detect differences in secondary outcomes, and the possibility of statistical error cannot be excluded. Second, patient-related factors such as expectations regarding analgesia, variations in peripartum care, and differences in individual pain thresholds and contraction patterns may have influenced the results and introduced subjective bias. Third, our findings are specific to the regimen of 0.08% ropivacaine + 0.3 μg/mL sufentanil, administered at 40-min intervals using a 25G Whitacre needle. Different epidural protocols, drug concentrations, or needle types may yield different results. Future studies with larger sample sizes covering the entire labor process and varied PIEB parameters are warranted to refine analgesic strategies for patients with GH.

## Conclusion

In summary, for patients with GH receiving DPE combined with PIEB (40-min bolus interval, 0.08% ropivacaine + 0.3 μg/mL sufentanil), the EV90 was 9.89 mL. This regimen achieved effective analgesia while reducing the risk of excessive sensory block and hypotension associated with higher bolus volumes (e.g., 12 mL), offering an evidence-based approach for optimizing labor analgesia in this high-risk obstetric population.

## Data Availability

The raw data supporting the conclusions of this article will be made available by the authors, without undue reservation.
